# Middle Age as a Window on Healthy Aging: The Midlife in the United States Study Over 30 Years

**DOI:** 10.1093/geroni/igaf122.1535

**Published:** 2025-12-31

**Authors:** Margie Lachman, David Almeida

## Abstract

The John D. and Catherine T. MacArthur Foundation provided 10 years of funding for the Research Network on Successful Midlife Development in 1995. The network included 12 scholars and 10 junior affiliates who designed the first wave of the Midlife in the United States (MIDUS) study. The initial study included a random digit dial sample of over 7000 adults ages 25 to 75 from the 48 contiguous United States. With funds from the National Institute on Aging, the study continues today with new samples added. MIDUS has garnered significant attention as the most frequently downloaded study at the National Archive of Computerized Data on Aging. Its extensive measures of biopsychosocial resources have highlighted resilience in the face of adversity as a central theme. In this symposium that marks the 30-year anniversary of MIDUS, the speakers, who have all been with the study since its inception, will present the history and rationale of the study along with recent innovations and some of the most intriguing findings that have shed light on the aging process. A longitudinal focus on early risk and protective factors during midlife is invaluable for optimizing health and well-being in later life. Almeida presents the history, design, and collaborative nature of the study. Mroczek discusses MIDUS as an exemplar of intergenerational science. Lachman highlights long-term effects of physical and psychosocial factors in midlife for later life health. Carr covers processes that contribute to social disparities in health. Ryff shows the importance of psychological well-being for health and longevity.

